# Study of photoluminescence property on cellulosic fabric using multifunctional biomaterials riboflavin and its derivative Flavin mononucleotide

**DOI:** 10.1038/s41598-019-45021-5

**Published:** 2019-06-18

**Authors:** Sweta Narayanan Iyer, Nemeshwaree Behary, Vincent Nierstrasz, Jinping Guan, Guoqiang Chen

**Affiliations:** 1 ENSAIT-GEMTEX, F-59100 Roubaix, France; 2 Université Lille Nord de France, F-59000 Lille, France; 3 Textile Materials Technology, Department of Textile Technology, Faculty of Textiles, Engineering and Business, University of Borås, SE-50190 Borås, Sweden; 4 College of Textile and Clothing Engineering, Soochow University, Suzhou, 215006 China

**Keywords:** Photochemistry, Biomaterials

## Abstract

Flavins are ubiquitous in nature and participate in various biochemical reactions mainly in the form of coenzyme Flavin mononucleotide (FMN) or as precursor such as Riboflavin (RF). Both flavins, RF and FMN are multifunctional bio-based molecules yielding yellow coloration and exhibit photoluminescence, UV protection, and redox properties. The aim of the present research study was to investigate the diffusion method as a technique to obtain photoluminescent cellulosic fabric using multifunctional RF and FMN. The photoluminescent moiety RF and FMN exhibited three maximum absorbance peaks at about 270 nm, 370 nm and 446 nm in aqueous solution at pH 7. The solutions of RF and FMN with concentration 4% and 20% (owf) at pH 7 were prepared and used in diffusion method for cellulosic fabric dyeing. The study involved the determination of color performance and evaluation of luminescence property of the dyed fabric using UV-visible spectrophotometer and photoluminescence spectroscopy, respectively. Under monochromatic UV lamp exposure emitting at 370 nm, the dyed fabric showed an intense emission of greenish yellow color, which was later confirmed by the intense photoluminescence observed at a wavelength of about 570 nm. The study demonstrates the theoretical evaluation of quantum efficiency (φ) obtaining maximum φ value of 0.28. Higher color strength value and improved wash fastness were obtained by treatment with different biobased mordants such as tannic acid and citric acid as well as calcium chloride for both RF and FMN. Additionally, ultraviolet (UV) protection ability for both RF and FMN dyed fabric were determined and showed UPF factor of 50+ and 35 respectively. The work allowed us to explore the photoluminescence property of riboflavin and Flavin mononucleotide for its application in the field of textiles as a new scope of producing photoluminescent textile along with multifunctional properties such as coloration and UV protection.

## Introduction

**Figure 1:**
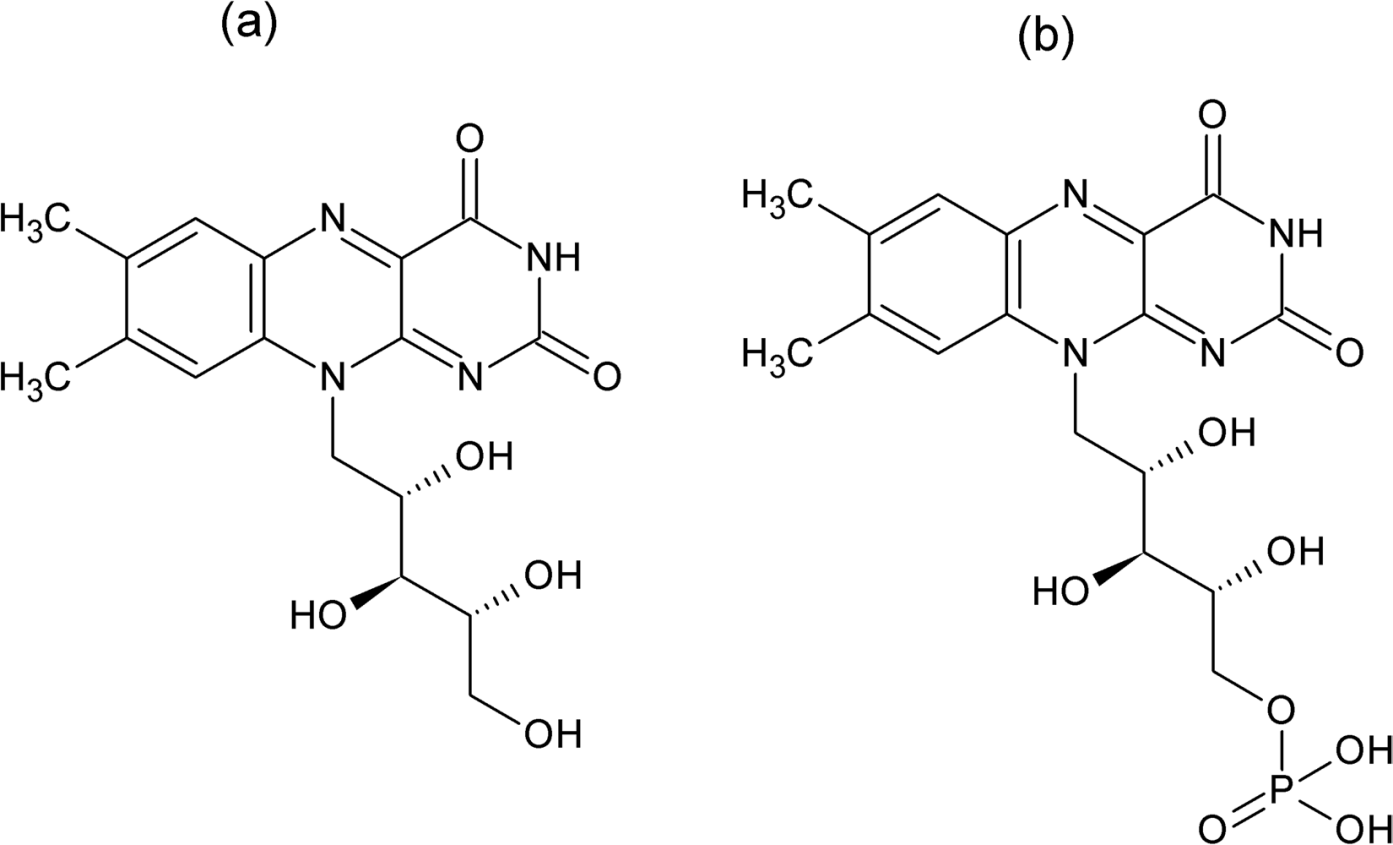
Chemical structure of (**a**) Riboflavin (RF) and (**b**) Flavin mononucleotide (FMN).

Riboflavin (RF) a biomolecule widely known, as Vitamin B2 is a naturally occurring compound and plays a vital role as a human nutrient^1^. It can be isolated from an extensive variety of animals and plants^2^ and its derivative Flavin Mononucleotide (FMN) is produced enzymatically using riboflavin as precursor^3^. Both RF and FMN possess distinctive biological and physicochemical property such as redox property, photosensitivity, and fluorescence property^4^. In contrast to riboflavin, which is sparingly soluble in water, FMN is highly water soluble due to the presence of ionic phosphate group. Further, the isoalloxazine ring in both RF and FMN is responsible for their fluorescence properties and exhibits strong absorption in both ultraviolet and visible regions^5^. Extensive studies on RF as a photosensitizer in food^6^ and in biological systems^7^ have been carried out while in the case of FMN, research work for its use as biosensors and bioengineering application fields have been published^8,9^. However, in the current work, we have explored the properties of riboflavin RF and its derivative FMN as a dye on cellulosic textile material using diffusion technique. In Fig. 1, structure formulae of (a) RF and (b) FMN are depicted.

Flavin mononucleotide (C_17_H_21_N_4_O_9_P) is a biomolecule produced from Riboflavin by an enzymatic reaction^10^. In the presence of oxidoreductase, the FMN also gets reduced into FMNH_2_, which is used for enzyme-catalyzed bioluminescence reaction^11^. Flavin mononucleotide is also used as an orange-red color additive for food and can be found in different food products such as milk products, jams, energy drinks, and sugar products^12^. Various studies on the photo, thermal and chemical degradation of both riboflavin and FMN have been carried out. Their fluorescence property, as well as corresponding quantum yields, have been widely studied^13^. The absorbance and fluorescence ability of riboflavin solutions can undergo changes or decrease in the peak intensity due to temperature effects and can vary depending upon the concentration^14^.

Luminescent materials have a wide range of applications in different industries as they have higher lightness and saturation. Application of luminescent materials include fields such as apparel, furnishings, printing and warning sign, safety alert or as a design feature in the interior, architectural as well as automotive textiles^15^. Since past few years light emitting materials are produced using different techniques such as LEDs, electro-luminescent wires, optical fibers, and electronic textiles to create light-emitting fabrics^16–18^. However, all these technology require electrical energy and possess high cost.

**Figure 2:**
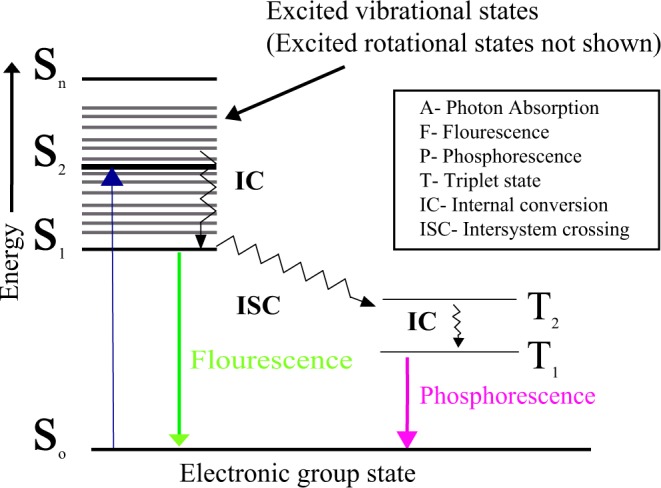
Jablonski diagram explaining photoluminescence, fluorescence and phosphorescence.

The term luminescence, in general, involves absorption of energy and subsequent emission of light. Fluorescence and phosphorescence are two broad forms of photoluminescence, based on absorption and re-emission of the photon as seen in Fig. 2.

In fluorescence, the emission of a photon is rapid while in the case of phosphorescence, the light emitted is delayed. Indeed, photoluminescent pigments used to produce photoluminescent textiles fall onto the category of “Glow in Dark”. The concept of “Glow in Dark” is the process of light emission when a fluorescent dye absorbs blue light emitted either by a phosphorescent material or by UV light from the atmosphere (example. Sunsets, sunrise and UV lamp). Photoluminescent textiles are also obtained either by fixing pigments in the fiber or onto the polymer fibrous surface using chemical binders by coating or printing producing glow in dark patterns on textiles. Accordingly, they can give textile a softer quality by emitting light without using any wires. Presently, photoluminescent molecules or particles such as rare earth doped Strontium aluminate (SrAl_2_O_4:_ Eu^2+^, Dy^3+^) used to produce photoluminescent textiles are high-cost rare earth luminescent and seem to have health impacts on workers^19–21^. It is, therefore, necessary to find alternative bio-based luminescent molecules to obtain photoluminescent textiles. Riboflavin is a potential biomolecule and to our knowledge, until now no work has been carried out to determine the photoluminescence property on textile substrate. Both Riboflavin and Flavin mononucleotide was selected as a fluorophore for the photoluminescence study on cellulosic fabric using diffusion technique. The study involved the determination of color performance and evaluation of luminescence property of the dyed fabric using UV-visible spectrophotometer, monochromatic UV lamp (emitting at 370 nm) and photoluminescence spectroscopy. In addition to the determination of photoluminescence property using spectroscopy methods, quantum efficiency was calculated theoretically on textile fabric. The influence of biomordants on the dyed fabric was assessed. The aim of the work was to explore the photoluminescence property of Riboflavin and Flavin mononucleotide for their application in the field of textiles producing photoluminescent textile along with multifunctional properties.

## Experimental

### Materials and chemicals

Cellulosic woven material such as viscose fabric 40 g/m^2^ was primarily used and most of the evaluation of different functional properties was carried out on viscose fabric, however, cotton fabric of 70 g/m^2^ was also studied. Prior to dyeing, both viscose and cotton fabric samples were cleaned using non-ionic detergent with subsequent hot and cold-water wash treatment.

All chemicals such as riboflavin, riboflavin 5′-monophosphate sodium salt hydrate widely known as flavin mononucleotide (FMN), sodium phosphate monobasic dehydrate and sodium phosphate dibasic dehydrate were purchased from Sigma Aldrich and used as received without any further pre-treatment.

### Diffusion method

**Figure 3:**
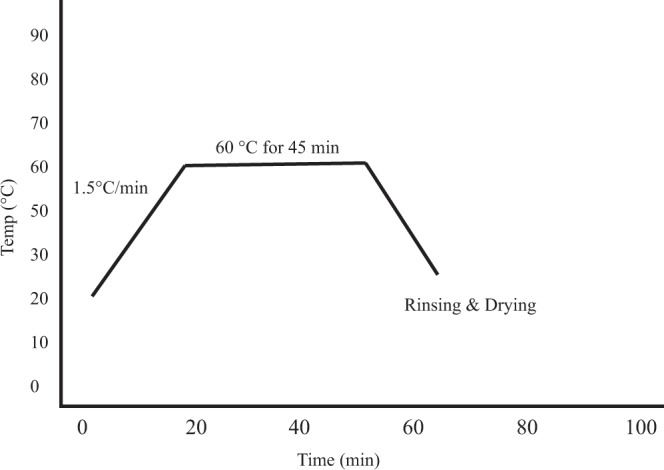
Condition for dyeing of textile using RF and FMN.

Pre-cleaned fabric samples were dyed in 200 ml beakers using the diffusion technique in a HTHP (High Temperatue and High Pressure/Beaker Dyeing Machines) as depicted in Fig. 3. Dye solutions of 4% and 20% of the weight of the fabric (owf) were prepared using phosphate buffer at pH 7. The temperature of the exhaustion bath was set to rise at about 1.5 °C/min up to 60 °C and maintained at this temperature for about 45 min. The dye bath was then cooled down to 45 °C temperature and the dyed fabrics were air dried at ambient temperature overnight. In the process, replicated dyed fabrics of 4% and 20% owf were generated for assessment of properties for both washed and unwashed fabric samples after dyeing. The dyed fabrics were rinsed and washed using warm and cold water subsequently. The treated fabrics were assessed for color strength value (K/S) and other functional properties such as photoluminescence and UV protection ability.

### Evaluation of coloration

The color strength of the dyed fabrics was evaluated using a Konica-Minolta CM3610A spectrophotometer (illuminant D65, 10° standard observer). The measurement data provided the K/S, L*, a*, b* color coordinates which are lightness (L*), redness-greenness value (a*), yellowness-blueness value (b*). Consequently, to describe the depth of color on the textile substrate Kubelka-Munk theory was used (eq. 1). Using the equation (1) below, the K/S value was determined from reflectance values (R) measured for different wavelengths varying between 360 to 740 nm,

1 $$\frac{K}{S}=\frac{{(1-R)}^{2}}{2R}$$

where R is the diffused reflectance, K is the absorption coefficient of the sample and S is the scattering coefficient.

### Experimental set up to observe illumination of dyed samples under UV lamp

**Figure 4:**
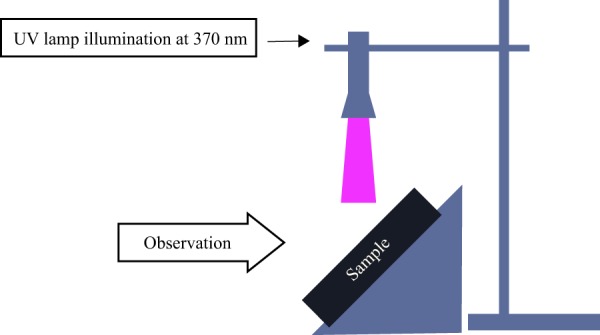
UV set up for measurement of treated samples.

The dyed fabrics were exposed to a monochromatic UV lamp emitting at 370 nm using the set up as seen in Fig. 4. The light emitted by the fabric in the form of fluorescence was captured using a camera.

### Photoluminescence spectroscopy

The photoluminescence emitted by the fabric sample was analyzed by a triple monochromator Dilor RT 30 and then detected using a Hamamatsu R943 photomultiplier in the photon-counting mode. Photoluminescence spectra were recorded at room temperature and the emission spectra were obtained by scanning with the analyzing spectrometer using fixed illumination at wavelengths of 364 nm and 470 nm line with 30 mW argon laser. The laser beam was focused with a 100-mm focal length lens to produce a 50-micron diameter with an average exposure time of 4 seconds spot on the sample and the incident power on the fabric sample was kept at 3 Mw.

### Quantum efficiency measurement

In addition to spectroscopy analysis measuring the emission spectra and photoluminescence intensity, the photoluminescence property was further investigated theoretically by quantifying the quantum efficiency of the dyed samples. The method involves measurement of spectral reflectance curve obtained using illuminant D65, 10° standard observer in Konica-Minolta CM3610A spectrophotometer. The spectral reflectance measurements of the dyed samples along with the undyed sample were recorded. The measured values were substituted in extended Kubelka Munk^22,23^ equation (2) stated below and quantum efficiency value was obtained respectively.

2 $${R}_{f(\lambda ^{\prime} )}=\frac{{K}_{f}}{S}\phi (\lambda ^{\prime} )\frac{(1+{R}_{\infty (\lambda ^{\prime} )})(1+{R^{\prime} }_{\infty })}{(\frac{1}{{R}_{\infty (\lambda ^{\prime} )}}-{R}_{\infty (\lambda ^{\prime} )})+(\frac{1}{{R^{\prime} }_{\infty }}-{R^{\prime} }_{\infty })}$$

3 $$\frac{{K}_{f}}{S}=\frac{{(1-{R}_{\infty (\lambda ^{\prime} )})}^{2}}{2{R}_{\infty (\lambda ^{\prime} )}}-\frac{{(1-{R}_{S(\lambda ^{\prime} )})}^{2}}{2{R}_{S(\lambda ^{\prime} )}}$$

where,

*λ*′ is absorption wavelength

$\phi (\lambda ^{\prime} )$ is quantum efficiency at *λ*′ wavelength

${R}_{\infty (\lambda ^{\prime} )}$ is spectral reflectance of the treated sample at *λ*′ wavelength

${R^{\prime} }_{\infty }$ is spectral reflectance of substrate at *λ*′ wavelength

*R*_*f*(*λ*′)_ is the difference in spectral reflectance value between forward and reverse measurement at the absorption wavelength

*K*_*f*_ is the absorption coefficient of the dyed samples minus that of the undyed samples

*S* is the scattering coefficient of the undyed sample in the emission region.

### UV protection ability

UV protection performance was evaluated for the dyed fabric samples. Labsphere UV 1000F ultraviolet transmittance analyzer (USA) was used to determine the ultraviolet protection factor (UPF) and UV transmittance of sample. Each sample was tested in quadrate and the obtained results were then averaged. The measurements were conducted according to the standard GB/T 18830-2009. Transmission measurement was evaluated in the range of 250–450 nm and consequently, the UPF ratings for all the treated fabric samples were obtained. The UPF value can be calculated using the following equation (4),

4 $$UPF=\frac{{\sum }_{250\,nm}^{450\,nm}\,E(\lambda )S(\lambda ){\rm{\Delta }}\lambda }{{\sum }_{250\,nm}^{450\,nm}\,E(\lambda )S(\lambda )T(\lambda ){\rm{\Delta }}\lambda }$$

where E (λ) is the relative erythemal spectral effectiveness, S (λ) is solar spectral irradiance, T (λ) is the spectral transmittance of each fabric and Δ λ is the wavelength step in nm.

## Results and Discussions

### UV/visible absorbance measurements

**Figure 5:**
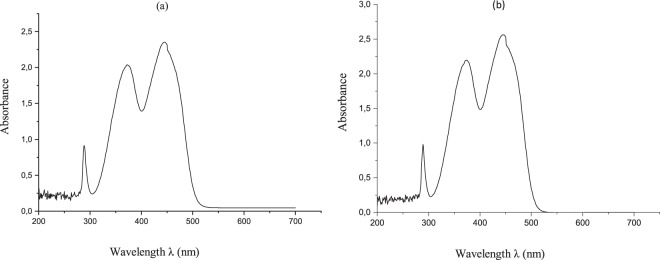
UV Visible spectroscopy of RF (**a**) and FMN (**b**) solution.

In Fig. 5, the UV-Visible spectra of 2 × 10^−4^ M RF and FMN solutions are depicted, with absorption plotted against wavelength ranging from 200 to 700 nm at room temperature. Similar absorption peaks obtained for both RF and FMN solutions with maximum peaks at 270, 373 and 444 nm for RF solution and 271, 374 and 445 nm for FMN solution. The RF aqueous solution was dark yellow in comparison to the appearance of FMN, which was a transparent yellow color. However, both showed bright yellow-green fluorescence under exposure to UV light at 370 nm.

### Color performance

**Figure 6:**
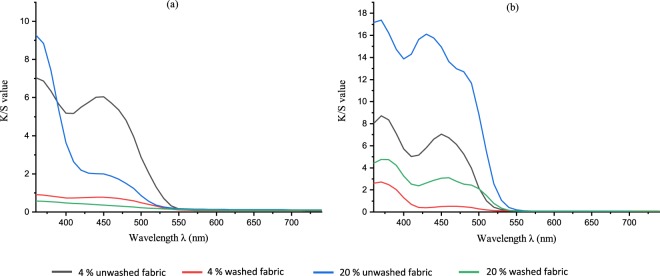
K/S value of viscose dyed fabric using 4% and 20% owf RF (**a**) and FMN (**b**).

**Figure 7:**
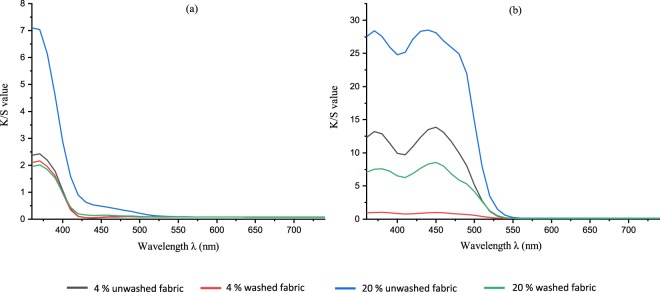
K/S value of cotton dyed fabric using 4% and 20% owf RF (**a**) and FMN (**b**).

**Figure 8:**
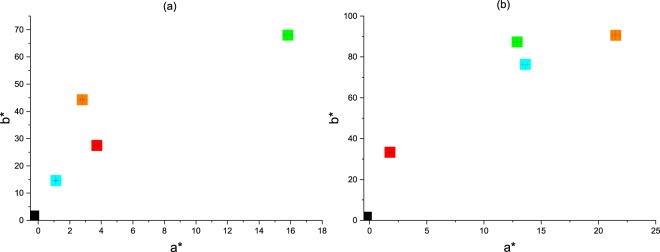
Color coordinate of 4% and 20% owf viscose dyed fabric samples  Undyed  4% washed fabric  4% unwashed fabric  20% washed fabric  20% unwashed fabric.

The colorimetric measurements of the dyed cellulosic fabrics were analyzed using the CIE Lab color space. The color performance was determined for both unwashed and washed treated fabric samples, as depicted in Figures 6 and 7. From the K/S spectral curves of the dyed fabrics, two main absorption peaks at around 360 to 370 nm and at 450 nm were observed, similar to absorption peaks observed in aqueous solutions. In the case of the FMN dyed fabric sample, the maximum color strength was observed at 370 nm, close to the absorbance of the aqueous FMN solution. For the RF dyed fabric sample, maximum color strength was observed at 360 nm, whereby a shift of 10 nm can be seen in comparison to the absorbance peak of aqueous riboflavin solution. Higher K/S values were obtained for the FMN dyed fabric, with a major increase in K/S value from 13 to 28 (at wavelength 370 nm) with an increase in dye owf from 4% to 20%. However, there was only a minor shift or increase in K/S from 7 to 9 observed for RF dyed fabric samples at wavelength 360 nm, as seen in Fig. 8. The color strength was also measured for cotton fabric showing K/S values of 2 and 7 for RF dyed samples at wavelength 360 nm along with K/S values of 8 and 17 for FMN dyed fabric samples at wavelength 370 nm for both 4% and 20% owf respectively. Overall, the color performance study revealed that FMN dyed viscose and cotton fabric samples possess maximum color strength at 370 nm, while the RF dyed, viscose, as well as cotton fabric, showed wavelength shift of 10 nm and maximum color strength was observed at 360 nm. The color coordinates depicted in Fig. 8 reveals that both a* and b* are positive. However, the b* coordinate value was greater than a* which shows that the yellow color predominates over red. All the treated fabric samples possess yellow coloration which appears to be the same color as that of the Flavin moiety. Hence, the photoluminescence behavior could be evaluated under UV exposure. Both molecules RF and FMN have the same isoalloxazine ring structure, however, the water solubility of FMN was higher due to the presence of phosphate group and hence the diffusion of FMN in cellulosic fabric was seen to be more predominant in comparison to RF.

### Observation of treated fabric sample under UV light

**Figure 9:**
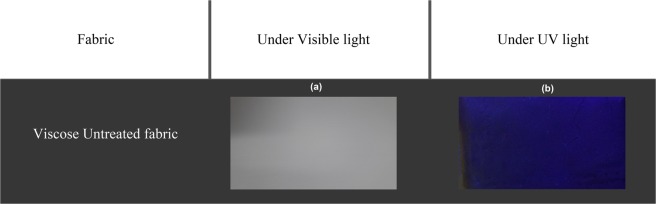
Image of Viscose untreated fabric under visible (**a**) and UV light (**b**).

**Figure 10:**
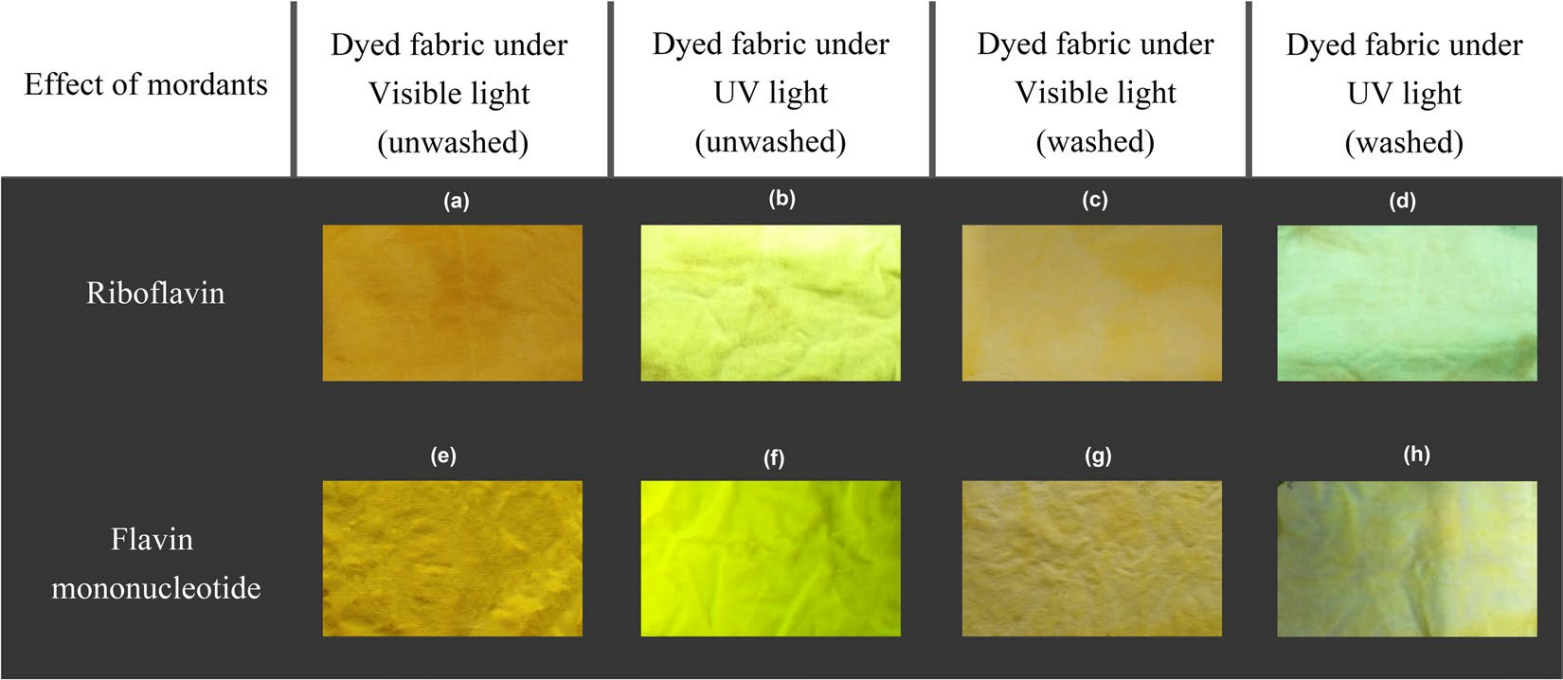
Images of Riboflavin (**a**–**d**) and FMN (**e**–**h**) viscose dyed fabric under visible and UV light.

**Figure 11:**
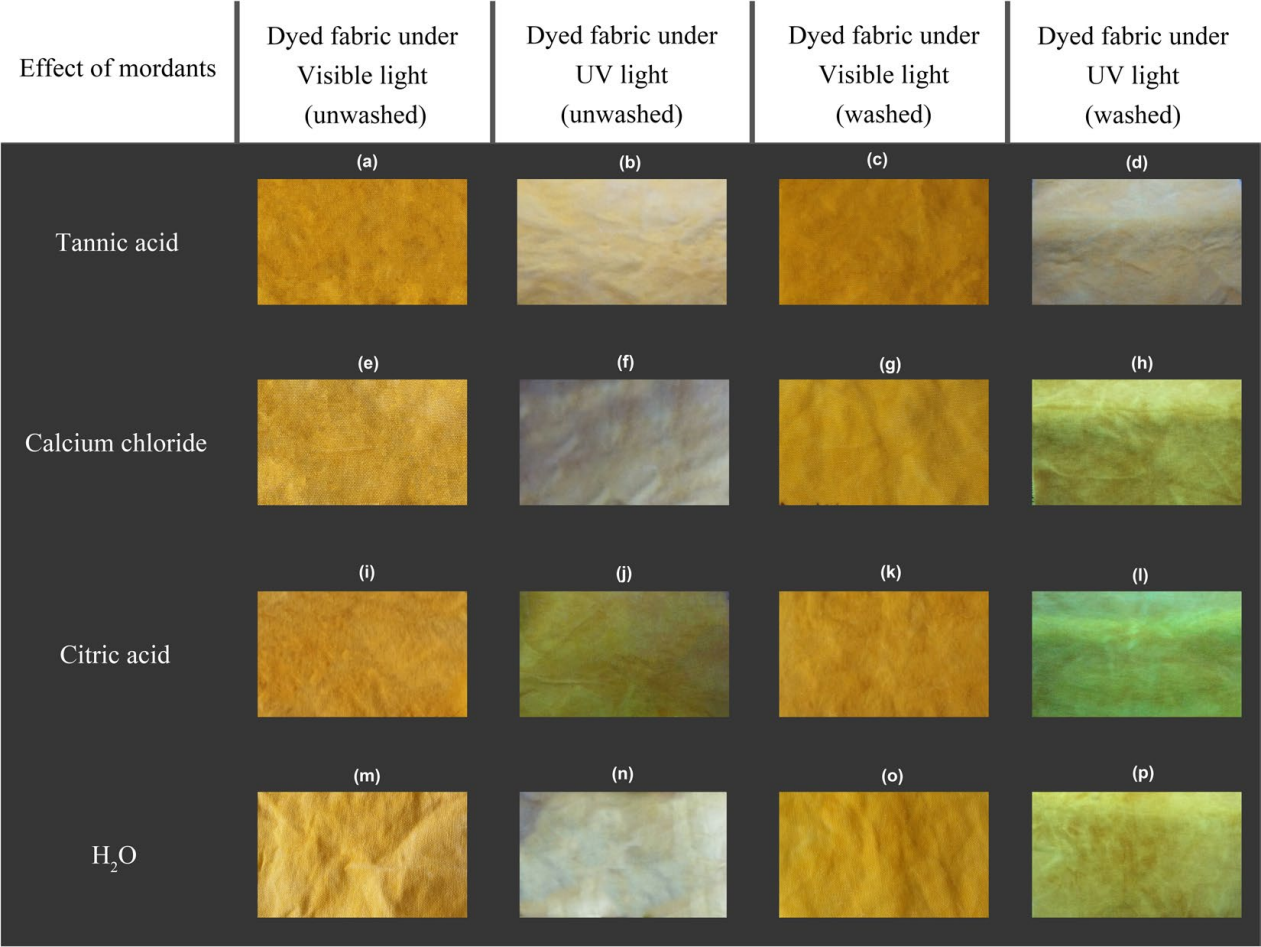
Images of Riboflavin dyed viscose fabric samples treated with tannic acid (**a**–**d**), calcium chloride (**e**–**h**), citric acid (**i**–**l**), H_2_O (**m**–**p**) as different mordants under visible and UV light.

**Figure 12:**
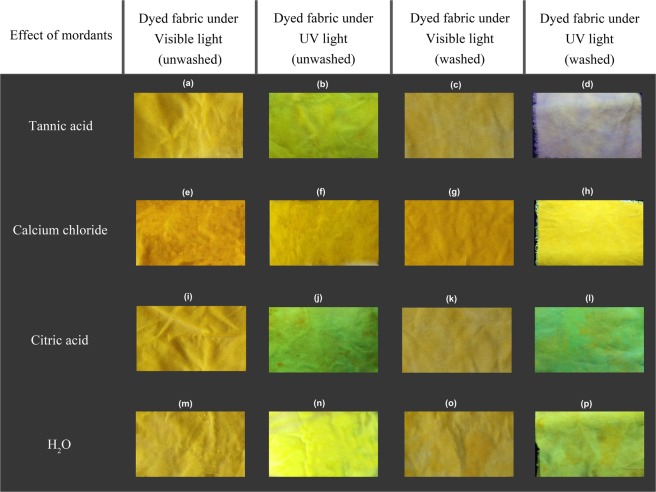
Images of FMN dyed viscose fabric samples treated with tannic acid (**a**–**d**), calcium chloride (**e**–**h**), citric acid (**i**–**l**), H_2_O (**m**–**p**) as different mordants under visible and UV light.

Figure 9 shows the pictures of untreated followed by pictures seen in the Figures 10, 11 and 12 of dyed samples illuminated with normal daylight and under monochromatic UV light of 370 nm. Intense yellowish green fluorescence was observed and markedly detectable by the naked eye in case of both RF and FMN dyed viscose fabric at 4% owf. As soon as the light source was removed, the dyed fabrics stopped illuminating, confirming the absence of phosphorescence phenomenon. In the presence of UV radiation, riboflavin and its derivative Flavin mononucleotide can absorb light energy to initiate to excited states and release energy in the form of visible light at higher wavelength, while the undyed viscose fabric illuminated with the same UV light at 370 nm exhibited no emitting color.

### Effects of mordants

**Figure 13:**
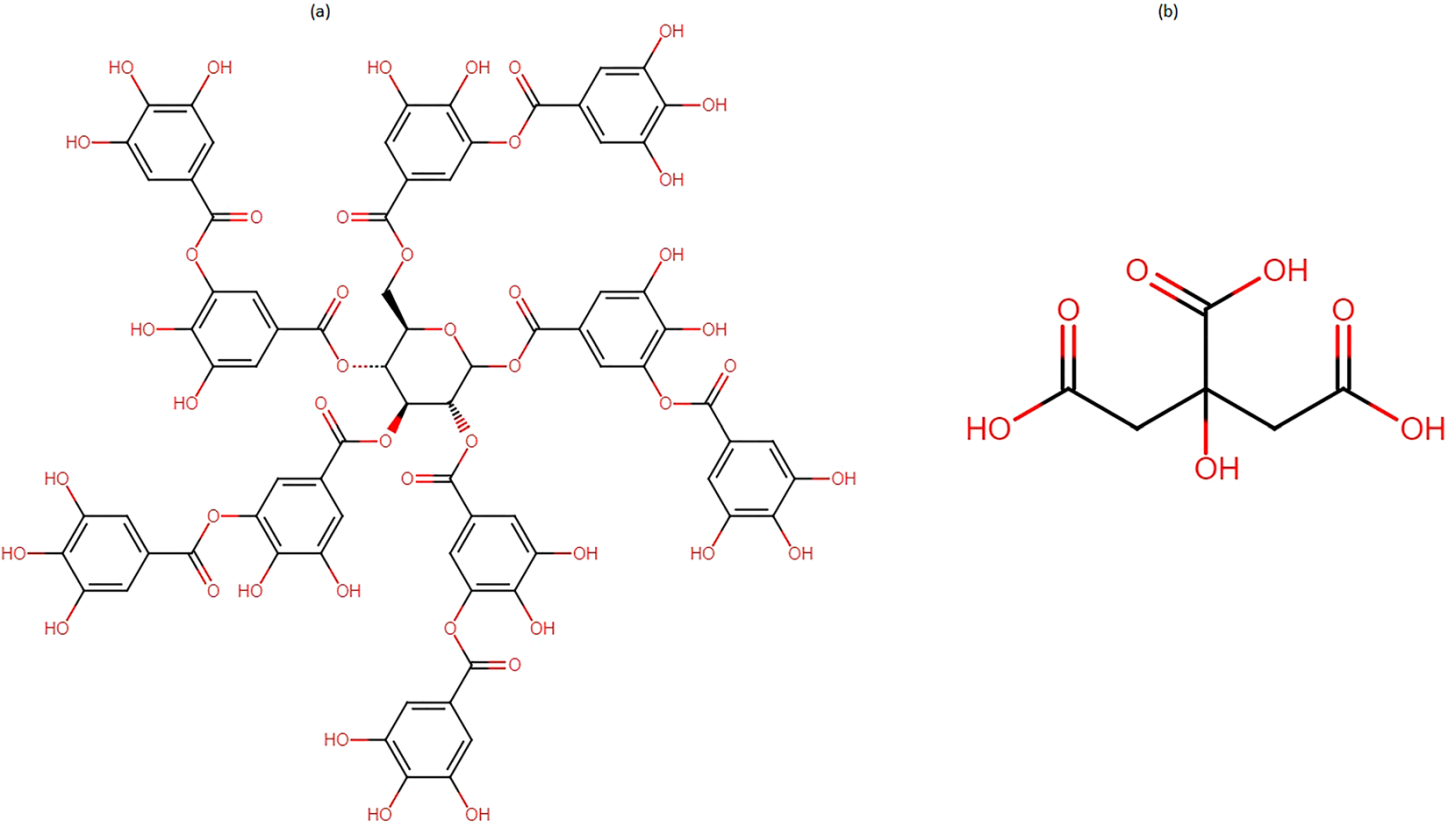
Structure of Tannic acid (**a**) and Citric acid (**b**).

The analysis of color strength property of both RF and FMN on cellulosic fabric showed decrease in K/S value after washings as expected and hence several biobased mordants were tested to improve the color strength value after washings. Different biobased mordants such as tannic acid and citric acid (structural formulae depicted in Fig. 13) as well as calcium chloride, which are nontoxic, were evaluated for the ability to enhance color strength value in 4% owf viscose dyed fabric. Mordanting was carried out after dyeing the samples by rinsing in 0.5% calcium chloride or 0.1% tannic acid or 0.5% citric acid. For comparison purpose, the dyed sample was also rinsed with water and the color strength of all the fabric samples was analyzed.

**Figure 14:**
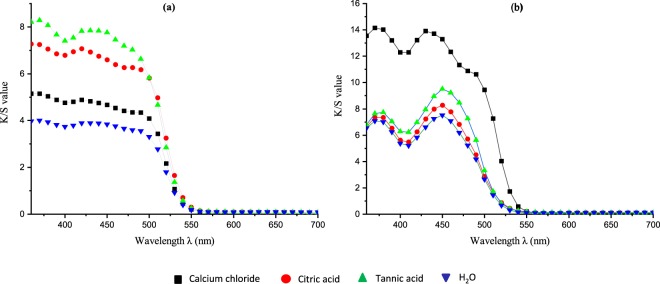
K/S value of unwashed viscose dyed fabric with different mordants using RF (**a**) and FMN (**b**).

**Figure 15:**
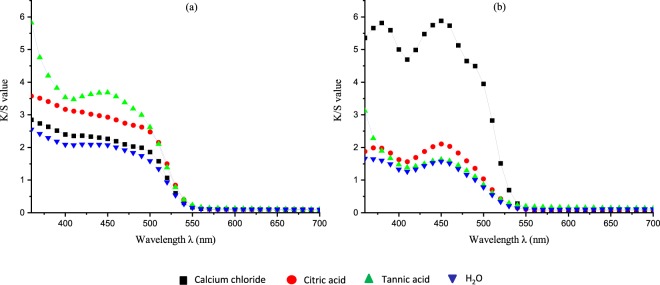
K/S value of washed viscose dyed fabric with different mordants using RF (**a**) and FMN (**b**).

In consideration of the RF dyed fabrics, the use of both tannic acid and citric acid mordants increased the color strength value with K/S value from 2 to 3 for citric acid and up to 6 for tannic acid (at 360 nm) as depicted in Figures 14 and 15, whereas calcium chloride had no major impact. In the case of FMN, calcium chloride yielded higher color strength and the K/S value increased from 1 to 5 (at 370 nm) as seen in Fig. 14. Further even citric acid mordanting led to slightly increased K/S values at both wavelengths of 450 nm and 370 nm followed by tannic acid mordanting which led to an increase K/S value at 350 nm only. Figures 11 and 12 shows the coloration of dyed fabric under normal daylight and under the UV light (370 nm). Yellowish fluorescence was seen for calcium chloride mordanted FMN dyed fabric samples. Citric acid and tannic acid mordanted FMN dyed fabrics also showed pale yellowish green fluorescence. Dyeing of cellulosic viscose using FMN with calcium chloride mordanting leads to the most probable occurrence of electrostatic binding of at least two FMN molecule. The stability in case of FMN dyed viscose fabric with calcium chloride as mordant is clearly governed by the metal ion affinity. The basicity of the negatively charged phosphate group (PO3^2−^) in FMN with the positively charged bivalent Ca^2+^ ion provides more stable binding of the dye complex to the cellulosic fabric. Fluorescence depicted in calcium mordanted dyed fabrics was yellowish in the case of the FMN and pale yellow fluorescence in the case of the RF. It is probable that the calcium ions did not bind to the RF, but the ions did probably fix to the viscose fiber^24^. Further investigations need to be carried out to understand this phenomenon. Indeed Tannic acid was able to bind proteins primarily through the formation of multiple hydrogen bonds between the phenolic groups of tannins and the carbonyl functions of the peptide linkages of proteins^25^. In case of dyeing of cellulosic viscose with RF along with tannic acid mordanting, numerous hydrogen bonding’s may occur between a tannic acid molecule and hydroxyl groups of the cellulose and of the RF dye. The use of citrate buffers influence the kinetics of photolysis for RF as well as FMN and decrease the fluorescence quenching leading to a stabilize RF solution^26^. Hence, the yellowish fluorescence can be seen even after wash for RF dyed fabric mordanted with citric acid (as seen in Fig. 11). However, the photostability of riboflavin solutions increases by introducing acids such as sulphuric along with different metal ions whereby the phenomena of decrease in photo destruction rate could occur due to protonation and formation of a complex in between the metal ions and the hydroxyl group of riboflavin respectively^27^.

### Photoluminescence

The photoluminescence of the dyed viscose fabrics was measured using photoluminescence spectroscopy to quantify the luminescence observed in the form of fluorescence under a UV lamp at 370 nm. Photoluminescence spectra were recorded at room temperature and the emission spectra were obtained using fixed illumination at two wavelengths of 364 nm and 470 nm line of a 30 mW argon laser. These were the only wavelengths available on the equipment used.

**Figure 16:**
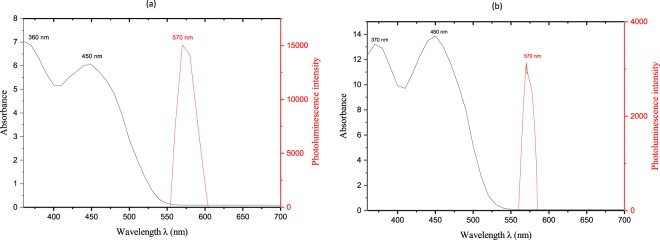
Absorbance of Riboflavin solution and the photoluminescence intensity data of Riboflavin dyed viscose fabric (**a**) and FMN dyed (**b**).

The luminescence spectra for both the dyed fabric using RF and FMN with 4% owf (unwashed) are illustrated in Fig. 16. Maximum photoluminescence intensity of the RF and FMN dyed viscose fabric was observed at an emission wavelength of 570 nm for both excitation wavelengths at 364 nm and 470 nm. Immediate photoluminescence at a higher wavelength of 570 nm confirmed fluorescence behavior of the dyed fabrics^28^.

Table 1 provides detailed photoluminescence values of 4% and 20% owf RF and FMN dyed viscose fabrics, for excitation wavelengths of 364 nm and 470 nm, before and after washing. For excitation at 470 nm, maximum photoluminescence at 570 nm as high as 22000 and 27000 au was recorded for the RF and FMN dyed fabrics, respectively. At this excitation wavelength (470 nm), the undyed viscose fabric also showed the value of 8000 au but at an emission wavelength of 560 nm. The undyed fabric also emitted at 500 nm at an excitation wavelength of 370 nm, with luminescence intensity 15000, which can occur due to use of optical brighteners in cellulosic fabric making process^29^ (as seen in Fig. 9). The intensity of dyed samples varied depending upon the photoluminescent moiety, concentration, and the excitation wavelength. At excitation wavelength of 364 nm, the FMN dyed fabric samples with 4% and 20% owf showed the intensity of about 9000 a.u and 11000 a.u respectively, however, there was an increase in the intensity value observed for the washed samples. At excitation wavelength of 470 nm, the intensity value observed was 27000 a.u for FMN (4% owf) and 19000 a.u for FMN (20% owf) which indicates a decrease in intensities with an increase in FMN concentration. However, the intensity decreased further for the FMN treated washed samples at excitation wavelength 470 nm. In case of RF dyed fabric samples, the intensity value remained the same at about 22000 even after the increase in concentration from 4% to 20% owf at both the excitation wavelength of 364 nm and 470 nm. The intensity value corresponds to maximum emission energy absorbed by the sample which emits back at 570 nm. A shift of fluorescence to higher wavelength (570 nm) compared RF and FMN aqueous solution (530 nm) may be explained by the excitation wavelengths (364 nm and 470 nm) which are different from the wavelengths of maximum absorption peaks of the RF and FMN aqueous solution (350–370 nm and 444–445 nm as seen in Fig. 5). The stimulated intensity observed at a higher wavelength of 570 nm for the fabric samples might be due to the intensity spectrum and energy difference of the laser beam which also lead to narrow emission peak^30^. The increase in the concentration of RF influences the fluorescence intensity where ionic strength log k quenches the fluorescence intensity^31^. As the concentration of riboflavin solution increased there was a decrease in the intensity resulting in quenching of fluorescence^27^, however, the riboflavin dyed fabric samples did not result in a decrease in intensity but remained almost equivalent even with the increase in concentration. Depending upon the mechanism of monomer-dimer energy transfer for FMN in water along with steady-state and time-resolved technique, there exhibit a decrease in fluorescence intensity decay with an increase in the concentration of FMN solution at λ excitation = 473 nm^32^. The FMN dyed fabric sample also showed a decrease in fluorescence intensity with increase in the concentration of FMN at an excitation wavelength of 470 nm.

**Table 1:** Photoluminescence intensity values for RF and FMN dyed fabric at a respective excitation wavelength.

Excitation wavelength (nm)	364	470
**Sample description**		
Viscose Undyed fabric	15000^□^	8000^#^
4% FMN dyed (unwashed)	9000	27000
4% FMN dyed (washed)	12000	15000
20% FMN dyed (unwashed)	11000	19000
20% FMN dyed (washed)	17000	10000
4% RF dyed (unwashed)	22000	19000
4% RF dyed (washed)	23000	22000
20% RF dyed (unwashed)	26000	25000
20% RF dyed (washed)	23000	18000

(Photoluminescence results based on data analysis with proper baseline detection).

^□^Intensity observed at λmax 500 nm, ^#^Intensity observed at λmax 560 nm.

According to the Stokes law, for a molecule to have fluorescence, it must first absorb the radiation and generally, only 5 to 10% of molecules that absorb the radiation eventually exhibit the fluorescence phenomenon. The Jablonski diagram provides an explicit representation of some processes involved in electronic energy transitions of the molecule. The wavelength of emitted radiation is independent of the exciting wavelength. Fluorescence intensity and concentration are co-related, wherein the fluorescence quenching phenomenon occurs at higher concentration thereby decreasing the intensity. Basically, there are three major factors influencing the quenching of fluorescence such as quantum efficiency, the intensity of incident radiation and molar absorptivity.

RF and FMN exhibit the fluorescence property, the absorbed light promotes the molecule to the excited singlet state and then excited state eventually return to the ground state by emitting fluorescence. The undyed viscose fabric showed maximum intensity at 500 nm for excitation wavelength 364 nm. Although the photoluminescence intensity was measured at two wavelengths 364 nm and 470 nm, the maximum intensity observed for both RF and FMN dyed fabric samples was at 570 nm, which significantly correlates with the observation of fabric samples under UV light irradiation at 370 nm.

### Quantum efficiency

The photoluminescence phenomenon was confirmed by the measurement of spectroscopy data as seen in Table 1, wherein emitted radiation was observed at a longer wavelength than that of the impinged wavelength on the photoluminescent material also depicted in Fig. 16. As discussed in the Jablonski diagram, the molecule undergoes a chain of events, which includes different photophysical events such as internal conversion or vibrational relaxation, intersystem crossing, fluorescence and phosphorescence^28^. Among the different events, the study revealed the classical visual observation of fluorescence phenomena, wherein the material stopped emission of light immediately after the removal of UV light. In addition to spectroscopy analysis, fluorescence quantum efficiency on the textile surface was measured for the various concentration of dyed samples, which also pertains to the lifetime measurement of the fluorescence sample which was reported to be about 4.7 ns^28,33^. Fluorescence lifetime being the intrinsic property does not depend on the method of measurement. The fluorescence lifetime is independent of intensity measurement and concentration of fluorescence moiety. The lifetime function can be considered as state function due to its dependent factors such as excitation wavelength and the light exposure duration^28^.

Quantum efficiency (QE) can be measured briefly using two methods, the two-monochromator method and two-mode method or one-monochromator method. However, the use of two-monochromator method involves high-cost and is commonly inaccessible, thus, extended Kubelka Munk theory using one-monochromator method was used to measure the quantum efficiency^22,23,34^.

Quantum efficiency of riboflavin and flavin mononucleotide molecule has been reported and depending upon the solvent being water or ethanol, QE value varies from 0.24 to 0.32^5,35,36^. In this study, the quantum efficiency of molecule diffused in fabric samples was determined. The quantum efficiency value was evaluated at both the absorption wavelength of 360 and 470 nm respectively. With the assumption that the difference between the forward and reverse spectral reflectance being 0.1, as both the spectral reflectance band do not overlap, the quantum efficiency of dyed textile was calculated using extended Kubelka Munk theory and tabulated in Table 2. The values varied with the increase in the dye concentration as well as the washed fabric samples show an increase in quantum efficiency. In case of 4% RF and FMN dyed fabric samples, the unwashed samples showed QE value of about 0.13 and 0.14 respectively, while both RF and FMN washed sample showed an increase in quantum efficiency and 0.28 QE value was obtained for each. Further, quantum efficiency values at both the wavelengths 360 and 470 nm were calculated which was observed to be invariant for FMN and RF dyed fabric samples. However, only in the case of 20% RF dyed fabric (unwashed), there was a difference in the quantum efficiency values at the respective wavelength.

**Table 2:** Quantum efficiency values for RF and FMN dyed fabric at a respective excitation wavelength.

Excitation wavelength (nm)	360	470
Sample description	Quantum efficiency at emission wavelength 570 nm	
4% FMN dyed (unwashed)	0.127	0.128
4% FMN dyed (washed)	0.274	0.255
20% FMN dyed (unwashed)	0.131	0.131
20% FMN dyed (washed)	0.138	0.137
4% RF dyed (unwashed)	0.14	0.143
4% RF dyed (washed)	0.286	0.287
20% RF dyed (unwashed)	0.144	0.206
20% RF dyed (washed)	0.164	0.157

(Photoluminescence results based on data analysis with proper baseline detection).

**Figure 17:**
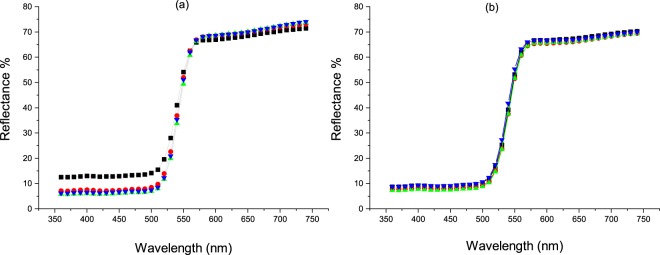
Spectral reflectance curve for RF dyed (**a**) unwashed and (**b**) washed fabric samples  4% owf  10% owf  16% owf  20% owf.

**Figure 18:**
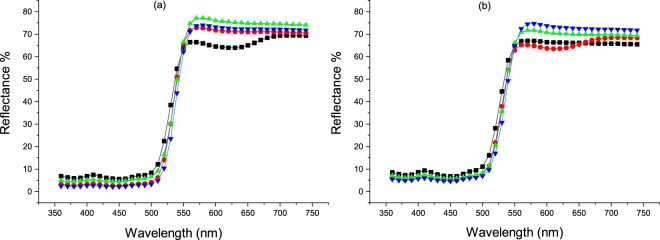
Spectral reflectance curve for FMN dyed (**a**) unwashed and (**b**) washed fabric samples  4% owf  10% owf  16% owf  20% owf.

Further studies were carried out and quantum efficiency of dyed textiles using different RF and FMN dye concentration were determined. In order to evaluate the effect of concentration on the quantum efficiency values which varied for 4% and 20% owf dyed fabric samples, dyeing was carried out at 4%, 10%, 16% and 20% owf respectively. Initially, the reflectance curves of the dyed textile fabric samples at different dye concentration were measured to calculate the quantum efficiency. As depicted in Figures 17 and 18 as the dye in the fabric has fluorescence moiety there was an increase in reflectance observed at wavelength 560 nm, where the photoluminescence intensity was also observed to be high as seen in Fig. 16.

The quantum efficiency for the dyed fabric samples at various concentration was calculated and values tabulated as in Tables 3 and 4. The quantum efficiency value of FMN as seen in Table 3 and RF dyed fabric in Table 4 revealed gradual increase in QE value with an increase in dye concentration from 4%, 10% to 16% (unwashed) samples and drops at 20% owf dyed fabric (unwashed sample). However, for the washed fabric samples the quantum efficiency was observed to be high at 0.28 for 4% owf, which then eventually dropped from 10% to 20% owf.

**Table 3:** Quantum efficiency at the various concentration for FMN dyed fabric samples.

Sample description	FMN dyed fabric (unwashed)		FMN dyed fabric (washed)	
Excitation wavelength(nm)	360	470	360	470
Dye %owf	Quantum efficiency calculated at a fluorescent emission wavelength of 570 nm			
4%	0.12897	0.12842	0.27386	0.25553
10%	0.13699	0.13947	0.15755	0.1562
16%	0.14723	0.14971	0.15428	0.14804
20%	0.12621	0.12755	0.15166	0.14253

**Table 4:** Quantum efficiency at the various concentration for RF dyed fabric samples.

Sample description	RF dyed fabric (unwashed)		RF dyed fabric (washed)	
Excitation wavelength(nm)	360	470	360	470
Dye concentration %owf	Quantum efficiency calculated at a fluorescent emission wavelength of 570 nm			
4%	0.14012	0.14342	0.28605	0.28793
10%	0.18036	0.19093	0.15755	0.16694
16%	0.14794	0.15606	0.15428	0.16335
20%	0.14224	0.15011	0.15166	0.16077

The average quantum efficiency for both 4% and 20% owf FMN dyed fabric sample shows 0.13, a significant increase in quantum efficiency can be seen for samples after washing in case of 4% owf dyed fabric, whereas 20% owf dyed fabric shows only minor increase. However, in the case of RF dyed fabric samples, a significant increase in quantum efficiency can be seen for both 4% and 20% owf dyed fabric samples.

**Figure 19:**
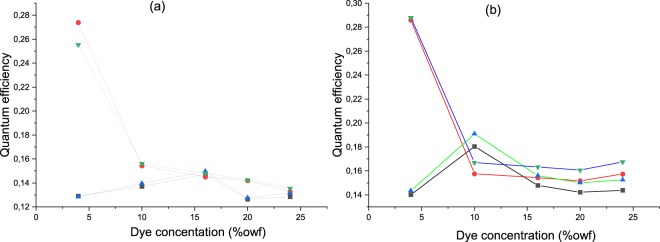
Quantum efficiency for RF dyed (**a**) and FMN dyed (**b**) fabric at different dye concentrations  QE @ 360 nm for dyed fabric (unwashed)  QE @ 360 nm for dyed fabric (unwashed)  QE @ 470 nm for dyed fabric (unwashed)  QE @ 470 nm for dyed fabric (washed).

The quenching concentration of riboflavin and flavin mononucleotide can be seen in Fig. 19, whereby the fluorescence quantum efficiency drops with the increase in dye concentration. As shown in Fig. 19, the fluorescence quantum efficiency varies depending upon the concentration of dye. The decrease in quantum efficiency value could occur due to molecule aggregation on the textile surface. The quenching concentration of riboflavin and FMN exist whereby the fluorescence quantum efficiency drops and the QE are invariant with the exciting wavelength. Hence, with the increase in the concentration of RF and FMN dye, quenching of fluorescence can be seen when measured the quantum efficiency, which drops at the concentration where the quenching effect begins. For both RF and FMN washed fabric samples the QE value drops from 10% and was seen to be almost stable having 0.15 QE value. In the case of RF and FMN dyed (unwashed) fabric samples the QE becomes stable from 16% having 0.14 QE value. The dyed fabric sample (unwashed) shows molecules physical adsorbed on the textile surface, as molecules were on fiber surface low quantity of reflectance was yielded and quantum efficiency was seen low. After washing, there was an increase in quantum efficiency due to the quenching effect with respect to the molecules on the surface and its effect in yielding fluorescence. Quantum efficiency can vary at different pH, the dyeing using riboflavin and FMN was carried out at pH 7 and hence the molecule could be rigid, thus the quantum efficiency was seen to be high^37^. Increase in rigidity yields an increase in fluorescence emission because of more conjugation in the isoalloxazine ring of RF and FMN molecule. Fluorophore has absorption spectrum which is also the excitation spectrum and in case of RF and FMN, the absorption was seen at wavelength 364 nm and 470 nm, whereas the wavelength at which the emission light obtained was at 570 nm and the maximum quantum efficiency value of 0.28 was determined.

### UPF factor

**Figure 20:**
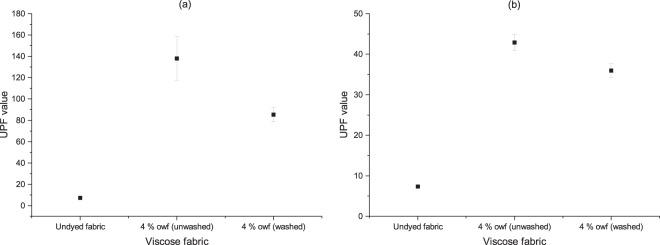
UPF factor of RF (**a**) and FMN (**b**) viscose dyed fabric samples.

Ultraviolet radiation ranges from near UV (290–400 nm), far UV (180–290 nm) and vacuum UV (below 180 nm). The term UVA represents the region 320–400 nm, UVB represents 290–320 nm and UVC region represents below 290 nm. The undyed viscose fabric showed a UPF value of 5, which reveals no effect to block UV radiation; however, the UPF rating of the dyed cellulosic fabric with both FMN and RF showed improved rating. The UPF value was greater than 50 for the RF dyed fabric samples and remained >50 even after wash which is highest when compared to FMN dyed fabric with UPF value of 35 and 30 for unwashed and washed fabric respectively as seen in Fig. 20. The FMN and RF absorption maximum can be seen in the visible region at 440 nm and 370 nm in UV region, hence improved UV protection effect were observed for both FMN and RF as seen in Table 5. Depending upon different factors such as physical/chemical type of fiber, fabric construction, thickness, and porosity, there may occur different type of interactions between the textile material and the ultraviolet radiation hitting the substrate affecting the ultraviolet protection ability^38^. The research study shows the impact of the UV rays on various living organisms such as premature aging and a major reason for malignant cutaneous melanoma, which is a skin disease. Studies have been carried out to cure the melanoma using RF^39^. Thus, the assessment provided the ultraviolet protection ability for both RF and FMN dyed fabric samples.

**Table 5:** UPF, UVA and UVB values of FMN and RF dyed fabric samples.

Sample Description	UPF	UV A	UV B
Viscose Undyed fabric	7.32	23.68	11.55
4% FMN dyed (unwashed)	42.92	2.08	2.28
4% FMN dyed (washed)	35.96	2.59	2.62
4% RF dyed (unwashed)	138.1	0.72	0.72
4% RF dyed (washed)	85.33	1.17	1.15

## Conclusion

The study confirms the potential use of diffusion method to impart photoluminescence on textiles along with multifunctional properties to the cellulosic fabric. Spectroscopic properties of Riboflavin or Flavin mononucleotide and it’s biological actions depend strongly upon the local environment, concentration and temperature; hence, it was important to detect the fluorescence property exhibited by the molecule on textiles. Most of the properties of both riboflavin and Flavin mononucleotide are transferred to the fabric as seen in the photoluminescence and UV protection data. In the case of FMN, high dye uptake was observed as seen in the K/S data when compared to RF treated fabric samples. Wash fastness increased using tannic acid and citric acid in case of riboflavin, however, tannic acid leads to a duller shade and less fluorescence when observed visually under UV illumination. Viscose fabric dyed using FMN and calcium chloride as mordant increases the wash fastness and the fluorescence property enhanced. The mordanting effect has to be further investigated to understand the phenomenon occurring between the RF and FMN moiety. The visual observance of photoluminescence allowed determining quantitatively the intensity of dyed samples. Also, theoretical evaluation of quantum efficiency on the textile fabric was carried out which revealed approximately the same quantum efficiency as that of RF and FMN molecule. It is well known, that the riboflavin solution exhibit antibacterial property, however, due to low deposition of molecules on the fabric samples the effect was not observed, as expected. Interestingly high UPF factors were observed for RF and FMN treated fabrics. The work allowed us to explore the photoluminescence property of riboflavin and Flavin mononucleotide for its application in the field of textiles as a new scope of producing photoluminescent textile in addition to other multifunctional property. However, further studies in case of dye fixation would be interesting in the perspective to obtain other functionalities such as antibacterial property along with the attainment and stability of photoluminescence effect on textiles.

## References

[1] Powers, H. J. Riboflavin (vitamin B-2) and health 1, 2. 1352–1360 (2018). 10.1093/ajcn/77.6.1352 12791609

[2] Galston A. W. (1950). Riboflavin, Light, and the Growth of Plants. Science.

[3] G.F.M. Ball. Flavins: Ribofl avin, FMN and FAD (Vitamin B 2). *B*. *CHAPTER* 165–175 (2006).

[4] Ghisla S, Massey V, Lhoste JM, Mayhew SG (1974). Fluorescence and Optical Characteristics of Reduced Flavines and Flavoproteins. Biochemistry.

[5] Kotaki A, Yagi K (1970). Fluorescence Properties of Flavins. J. Biochem..

[6] Huang R, Hyun JK, Min DB (2006). Photosensitizing effect of riboflavin, lumiflavin, and lumichrome on the generation of volatiles in soy milk. J. Agric. Food Chem..

[7] Makdoumi, K. *Ultraviolet Light A (UVA) Photoactivation of Riboflavin as a Potential Therapy for Infectious Keratitis*. (2011).

[8] Mertens ME (2014). FMN-coated fluorescent USPIO for cell labeling and non-invasive MR imaging in tissue engineering. Theranostics.

[9] Orita A, Verde MG, Sakai M, Meng YS (2016). A biomimetic redox flow battery based on flavin mononucleotide. Nat. Commun..

[10] Kearney, E. B. & Englard, S. The enzymatic phosphorylation of riboflavin. *The Journal of biological chemistry* **193**, 821–834 (1951). 14907770

[11] Inouye S (1994). NAD(P)H-flavin oxidoreductase from the bioluminescent bacterium, Vibrio fischeri ATCC 7744, is a flavoprotein. FEBS Lett..

[12] Martí-Andrés P., Escuder-Gilabert L., Martín-Biosca Y., Sagrado S., Medina-Hernández M.J. (2015). Simultaneous Determination of Pyridoxine and Riboflavin in Energy Drinks by High-Performance Liquid Chromatography with Fluorescence Detection. Journal of Chemical Education.

[13] Hustad S, Ueland PM, Schneede J (1999). Quantification of riboflavin, flavin mononucleotide, and flavin adenine dinucleotide in human plasma by capillary electrophoresis and laser-induced fluorescence detection. Clin. Chem..

[14] Sheraz MA, Kazi SH, Ahmed S, Anwar Z, Ahmad I (2014). Photo, thermal and chemical degradation of riboflavin. Beilstein J. Org. Chem..

[15] Kooroshnia, M. Designing a two-phase glow-in-the-dark pattern on textiles. *Shapeshifting A Conf*. *Transform*. *Paradig*. *Fash*. *Text*. *Des*. 1–16 (2014).

[16] Deckers, E. *Perceptive Qualities in Systems of Interactive Products*, 10.6100/IR753907 (2013).

[17] Liu Ya-Jun, De Vico Luca, Lindh Roland (2008). Ab initio investigation on the chemical origin of the firefly bioluminescence. Journal of Photochemistry and Photobiology A: Chemistry.

[18] Lim H (2010). A review of spun bond process. J. Text. Apparel, Technol. Manag..

[19] Ge M, Guo X, Yan Y (2012). Preparation and study on the structure and properties of rare-earth luminescent fiber. Text. Res. J..

[20] YU Yuan, WANG Jian, ZHU Yanan, GE Mingqiao (2014). Researches on preparation and properties of polypropylene nonwovens containing rare earth luminous materials. Journal of Rare Earths.

[21] Guo X, Zhang K, Zhang H, Ge M (2018). Working Conditions on the Afterglow Characteristics of Rare-earth Luminous Fibers. FIBERS Polym..

[22] Shakespeare T, Shakespeare J (2003). A fluorescent extension to the Kubelka-Munk model. Color Res. Appl..

[23] Rong L, Feng G, Jiangnin C, Donghui C (2002). Quantum efficiency of fluorescent dyes in cloth Coloration Technology. Color. Technol..

[24] Sigel H (1995). Acid-base and metal ion-binding properties of flavin mononucleotide (FMN2-). Is a ‘dielectric’ effect responsible for the increased complex stability?. Inorganica Chim. Acta.

[25] Bennick Anders (2002). Interaction of Plant Polyphenols with Salivary Proteins. Critical Reviews in Oral Biology & Medicine.

[26] Ahmad I (2011). Stabilizing effect of citrate buffer on the photolysis of riboflavin in aqueous solution. Results Pharma Sci..

[27] Astanov S, Sharipov MZ, Fayzullaev AR, Kurtaliev EN, Nizomov N (2014). Spectroscopic study of photo and thermal destruction of riboflavin. J. Mol. Struct..

[28] Berezin MY, Achilefu S (2010). Fluorescence Lifetime Measurements and Biological Imaging. Chem. Rev..

[29] Iqbal, I. & Rahman, M. M. Optimization of parameters of cotton fabric whiteness. (2014).

[30] Rivera JA, Eden JG (2016). Flavin mononucleotide biomolecular laser: longitudinal mode structure, polarization, and temporal characteristics as probes of local chemical environment. Opt. Express.

[31] Ahmad I (2016). Ionic strength effects on the photodegradation reactions of riboflavin in aqueous solution. J. Photochem. Photobiol. B Biol..

[32] Grajek H (2007). Flavin mononucleotide fluorescence intensity decay in concentrated aqueous solutions. Chem. Phys. Lett..

[33] Coppel G, Andersson M, Edström P, Kinnunen J (2012). Limitations in the efficiency of fluorescent whitening agents in uncoated paper. Nord. Pulp Pap. Res. J..

[34] Eckstein JW, Hastings JW, Ghisla S (1993). Mechanism of Bacterial Bioluminescence: 4a,5-Dihydroflavin Analogs as Models for Luciferase Hydroperoxide Intermediates and the Effect of Substituents at the 8-Position of Flavin on Luciferase Kinetics. Biochemistry.

[35] Salzmann, S. *et al*. Photophysical properties of structurally and electronically modified flavin derivatives determined by spectroscopy and theoretical calculations Supporting Information (SI) Molecular orbitals and vertical absorption energies in vacuum and different solve. **145**, 9365–9375 (2009). 10.1021/jp905724b 19639947

[36] Moore WM, McDaniels JC, Hen J (1977). A. the Photochemistry of Riboflavin—Vi. the Photophysical Properties of Isoalloxazines. Photochem. Photobiol..

[37] Reynolds GA, Drexhage KH (1975). New coumarin dyes with rigidized structure for flashlamp-pumped dye lasers. Opt. Commun..

[38] Saravanan, D. UV protection textile materials. *AUTEX Research Journal* **7**, 53–62 (2007).

[39] Machado D (2013). Irradiated Riboflavin Diminishes the Aggressiveness of Melanoma *In Vitro* and *In Vivo*. PLoS One.

